# Atypical Presentation of Fibrodysplasia Ossificans Progressiva: A Case Report and Review of Literature

**DOI:** 10.7759/cureus.2955

**Published:** 2018-07-10

**Authors:** Vivek Tiwari, Prateek Behera, Radha Sarawagi, Babu Mohammed Rafi, Saurabh Sahu, Hemanth Raj, Manish Rajpoot

**Affiliations:** 1 Department of Orthopaedics, All India Institute of Medical Sciences, Bhopal, IND; 2 Department of Radiodiagnosis, All India Institute of Medical Sciences, Bhopal, IND; 3 Department of Orthopaedic Surgery, All India Institute of Medical Sciences, Bhopal, IND

**Keywords:** fop, myositis ossificans progressiva, heterotopic ossification, fibrodysplasia ossificans progressiva, hallux valgus

## Abstract

Fibrodysplasia ossificans progressiva (FOP) is a rare genetic disease characterized by widespread areas of abnormal bone formation in muscles, ligaments, tendons and joint capsules. Typically, the symptoms begin in the first decade of life with episodes of painful inflammatory soft tissue swellings. Gradually, there occurs restriction of motion at various joints, severely limiting the activities of daily living and the quality of life of such patients by the third decade of life. There is no definite cure available for the disease and the current treatment options target symptomatic and palliative management. We describe the case of a 10-year-old child who presented to our institute with a severe disability of upper limbs due to joint contractures along with several bony masses at various locations of the body but without having any prior complaints of painful soft tissue lesions or the characteristic flare-ups of the disease ever. Identification of typical soft tissue ossified masses in the specific anatomic pattern, along with the presence of short and malformed great toes helped us in reaching the diagnosis. Surgical procedures including biopsies should be strictly avoided in such patients to prevent triggering the development of more lesions, which occurred in our patient after inadvertent removal of the first swelling by an orthopaedic specialist.

## Introduction

Fibrodysplasia ossificans progressiva (FOP), previously known as myositis ossificans progressiva, is a rare genetic disease characterized by widespread areas of progressive heterotopic endochondral ossification (HEO) [1]. Its incidence is nearly one per 2 million population worldwide [2]. FOP is a devastating disorder with significant cumulative disability due to the formation of an extra hard skeleton around the normal skeleton, giving this disease eponyms like “Stone man disease” [3]. Although such patients display certain tell-tale signs of the disease at birth such as bilaterally small great toes with hallux valgus [2] and abnormal calcaneal ossification [4], the condition is seldom diagnosed at birth. Typically, the symptoms begin in the first decade of life with repeated episodes of painful inflammatory soft tissue swellings, which later lead to the formation of hard bony masses in muscles, ligaments, aponeuroses and joint capsules [1]. Gradually, there occurs restriction of motion at various joints, severely limiting the activities of daily living and the quality of life of these patients by the third decade of life. There is no definite cure available for the disease and the current treatment options target symptomatic and palliative management [5]. We describe the case of a 10-year-old child with a severe disability of upper limbs due to joint contractures along with several bony masses at different locations of the body, without complaints of painful soft tissue lesions or flare-ups of the disease ever despite repeated questioning regarding episodes.

## Case presentation

A 10-year-old boy presented to our outpatient department with complaints of multiple swellings in the back along with swellings in both the arms since last eight years. He was born out of a normal vaginal delivery at term and the perinatal period was uneventful. His parents noticed deformity in both great toes at birth, without any other associated anomalies. The child attained all his developmental milestones without any developmental delay and was apparently alright till two years of age. At two years, his parents noticed a gradually increasing painless swelling on the posterior aspect of his left proximal arm associated with restricted extension of the left elbow joint. The child was operated for the swelling by an orthopaedic specialist, but the restriction in movements persisted and the swelling recurred.

**Figure 1:**
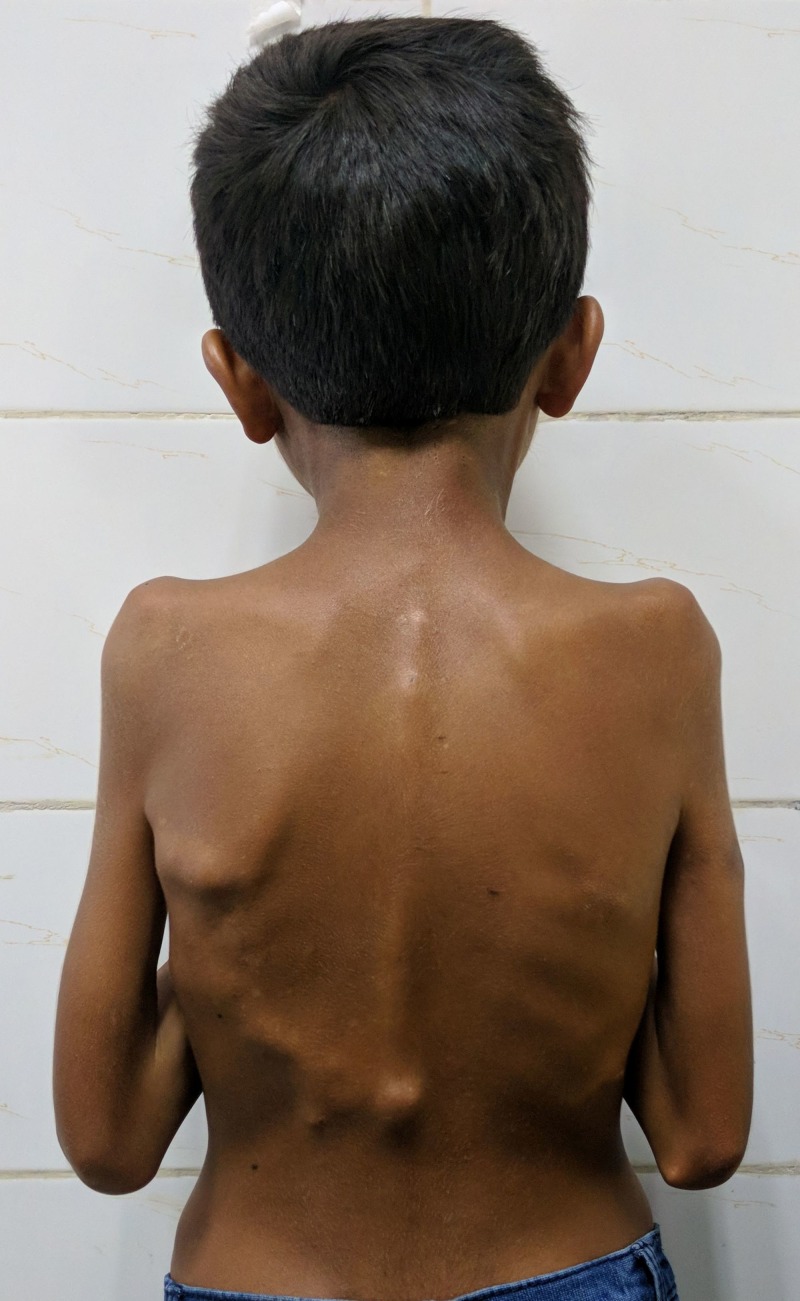
Clinical photograph of the back region. Multiple small, irregular, non-tender, bony hard swellings were present over bilateral infrascapular regions extending up to the lower dorsal spine.

Six months later, a gradually increasing painless swelling was noticed in the right arm, followed subsequently by restricted movements of the right elbow. Gradually, multiple swellings appeared over bilateral infrascapular regions. His shoulder movements also became restricted with time leading to severe limitation of his activities of daily living. At the presentation, the child had difficulty in feeding himself along with an inability to dress and undress. On examination, multiple small, irregular, non-tender, bony hard swellings were present over bilateral parascapular and infrascapular regions extending up to the lower dorsal spine (Figure 1).

**Figure 2:**
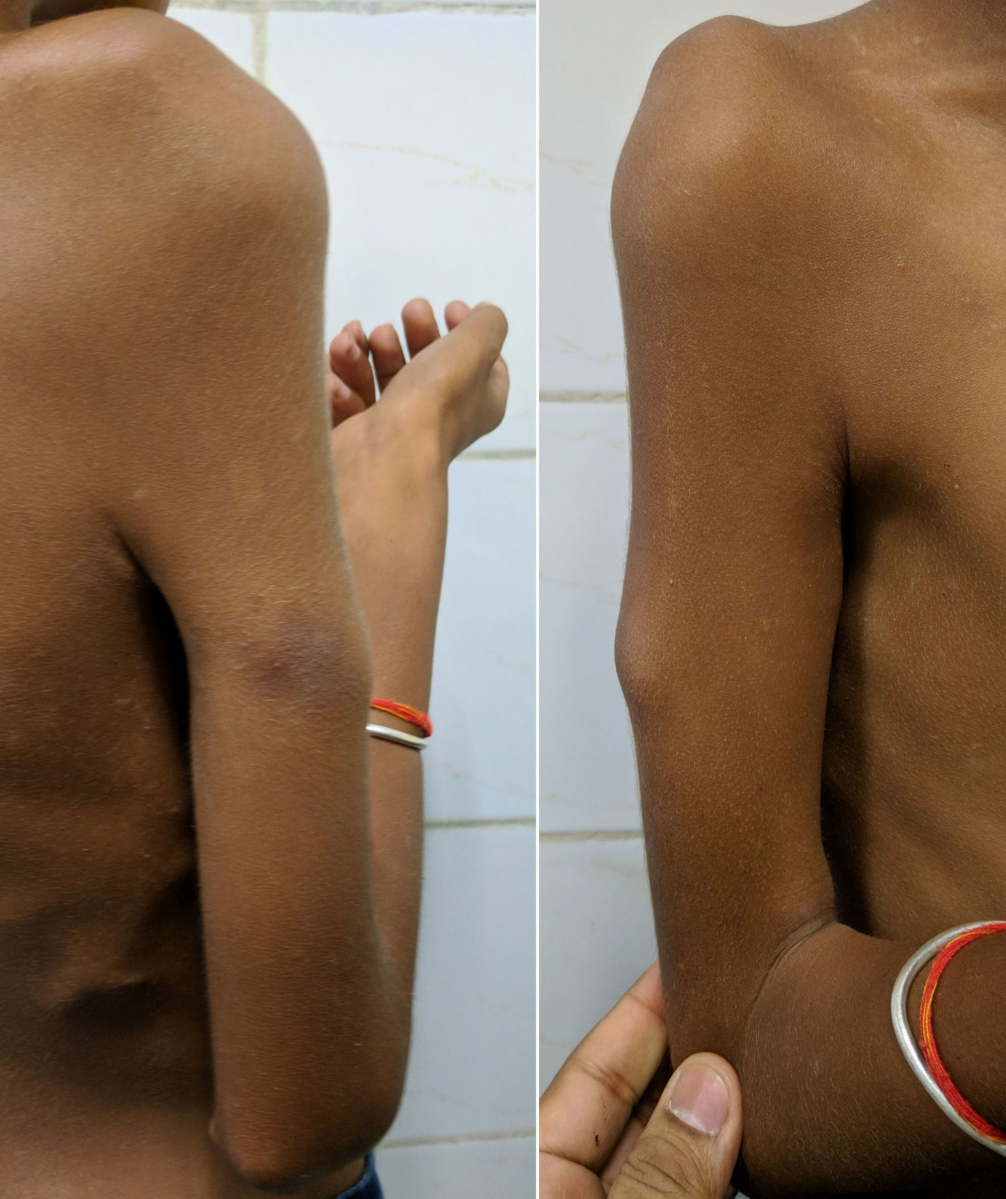
Clinical photograph of the right arm. Irregular, non-tender bony hard swelling was palpated in right arm associated with fixed flexion deformity of 90° at the elbow joint.

All the shoulder movements were found to be severely restricted bilaterally with 10° flexion and abduction movements. Non-tender bony hard swellings could be palpated in both the arms primarily on medial and posterior aspects, which were associated with fixed flexion deformity of 90° at both the elbow joints with a further 10° flexion movement possible (Figure 2).

**Figure 3:**
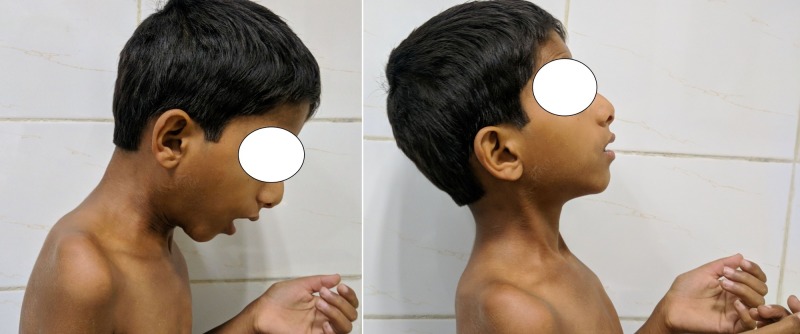
Clinical picture demonstrating restriction in neck movements. There was severe restriction of the neck flexion and extension movements.

Also, there was a severe restriction of the neck flexion and extension movements (Figure 3).

**Figure 4:**
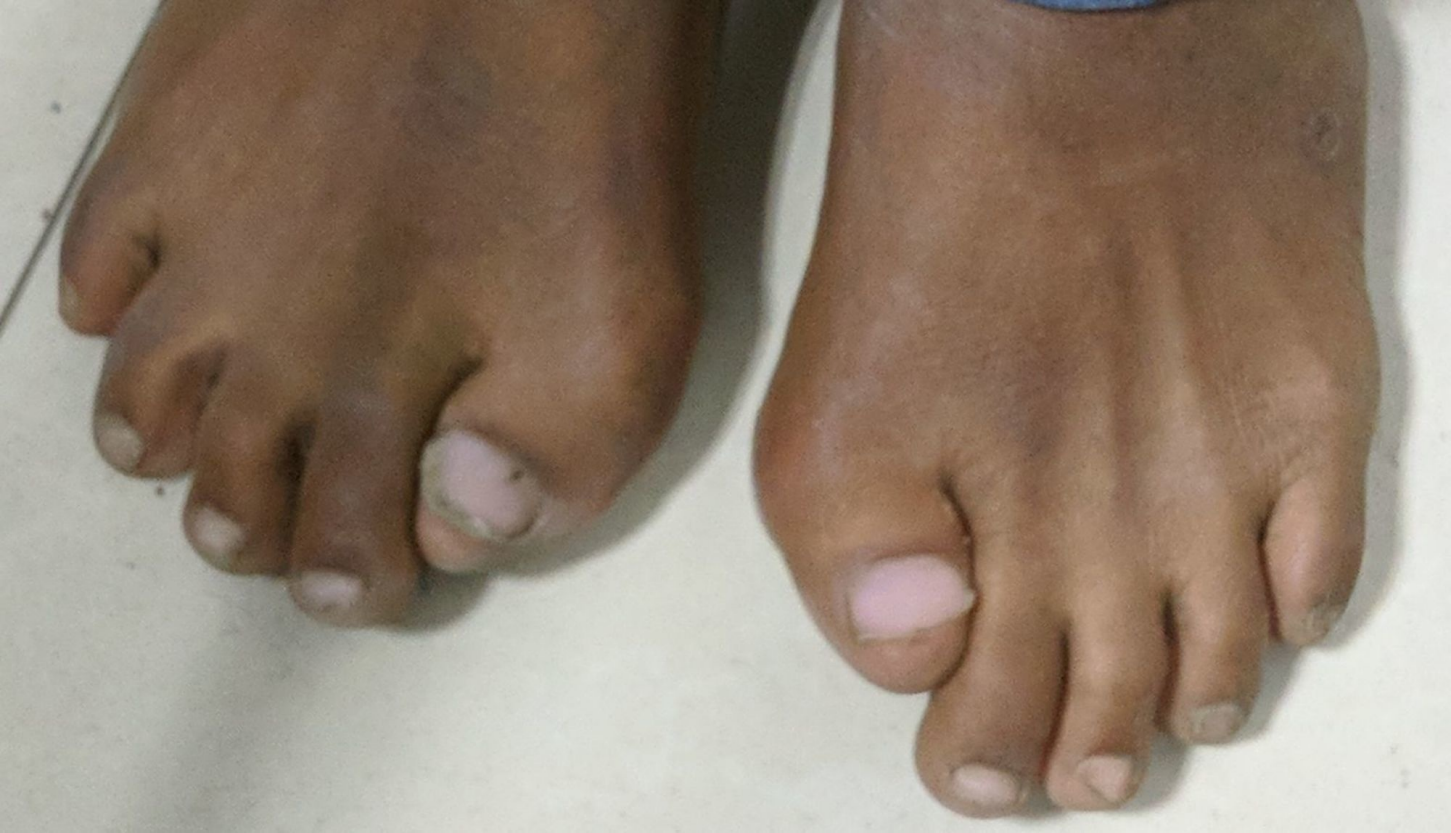
Clinical photograph of bilateral feet. Hallux valgus was present bilaterally along with microdactyly of the great toes.

The hip movements were also severely restricted on both sides allowing just up to 60° flexion causing restriction in sitting properly on a chair and inability to squat and sit cross-legged. Besides, there was a small, irregular, bony hard swelling palpable on the lateral aspect of the right distal thigh. On examination of the feet, there was hallux valgus present bilaterally along with microdactyly of the great toes (Figure 4).

Routine laboratory investigations including complete blood counts, erythrocyte sedimentation rate (ESR), C-reactive protein (CRP) and all other biochemical parameters were unremarkable.

**Figure 5:**
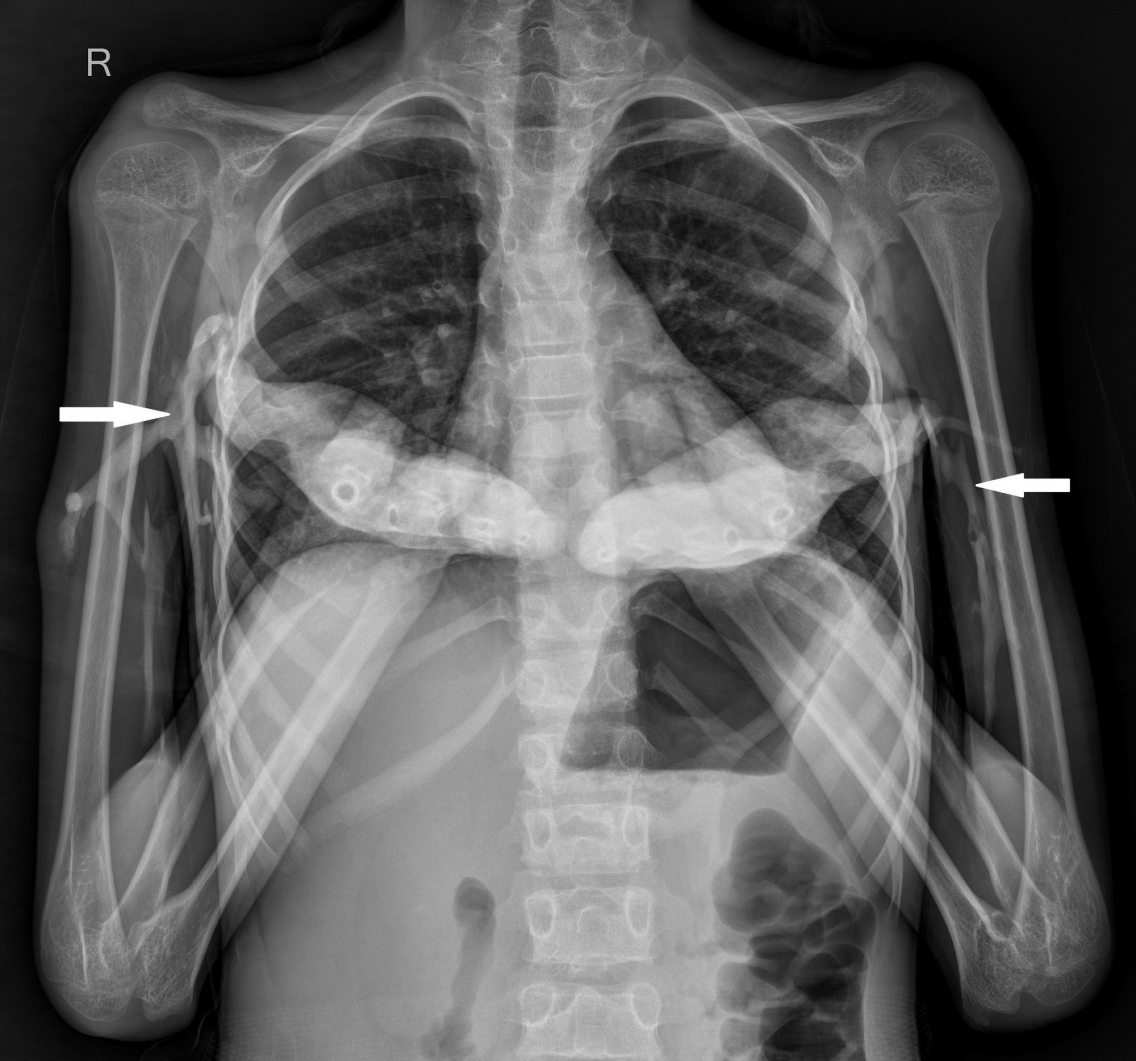
Plain radiograph of chest with bilateral arms - anteroposterior view. Heterotopic ossification was seen in the soft tissues around humerus on both sides (white arrows), extending through the axilla to the chest wall.

On plain radiographs of the chest with bilateral arms, heterotopic ossification was seen in the soft tissues around humerus on both sides, extending through the axilla to the chest wall (Figure 5).

**Figure 6:**
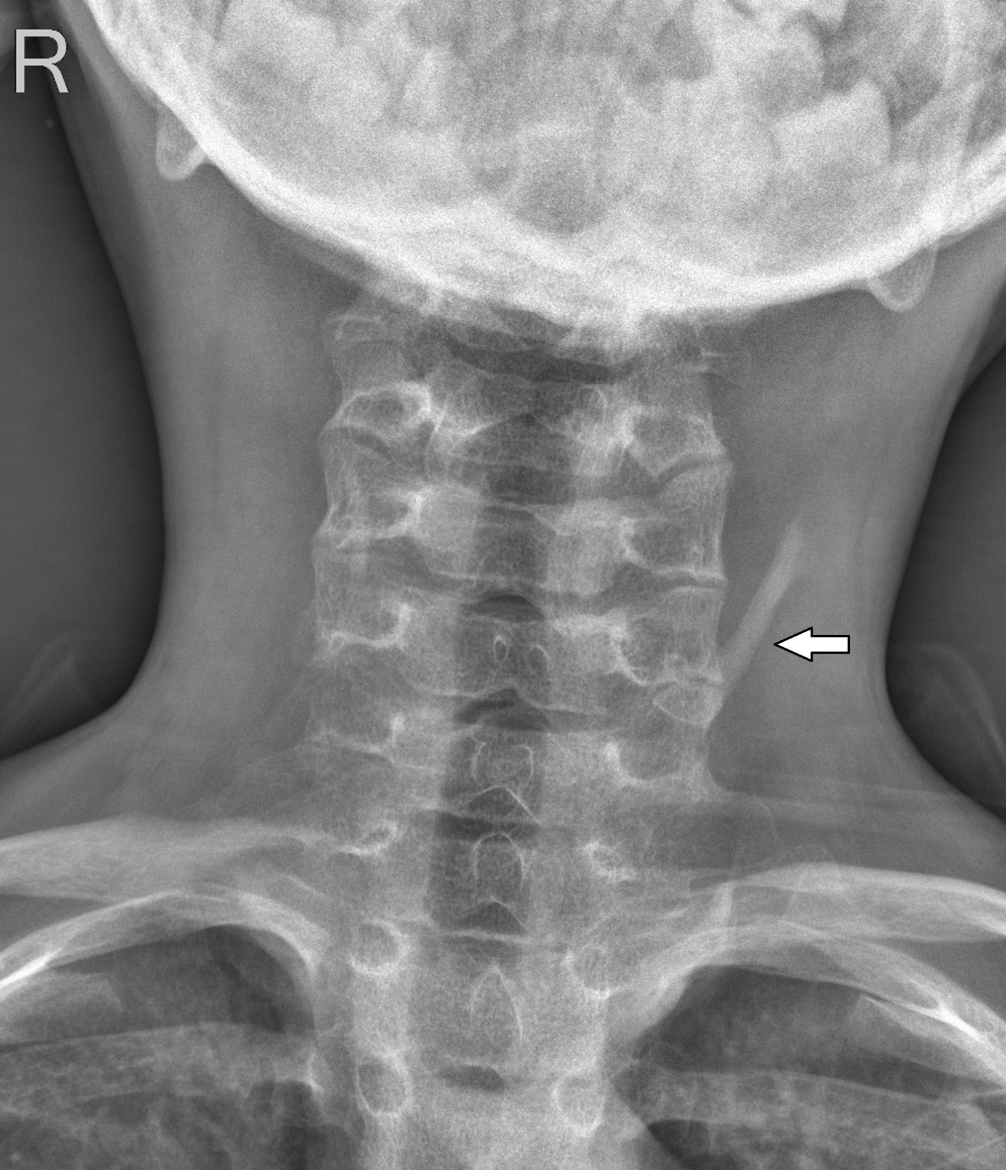
Plain radiograph of cervical spine – anteroposterior view. Heterotopic ossification was noted along the left side of neck (white arrows).

Heterotopic ossification was also noted along left side of the neck on radiograph of cervical spine (Figure 6).

**Figure 7:**
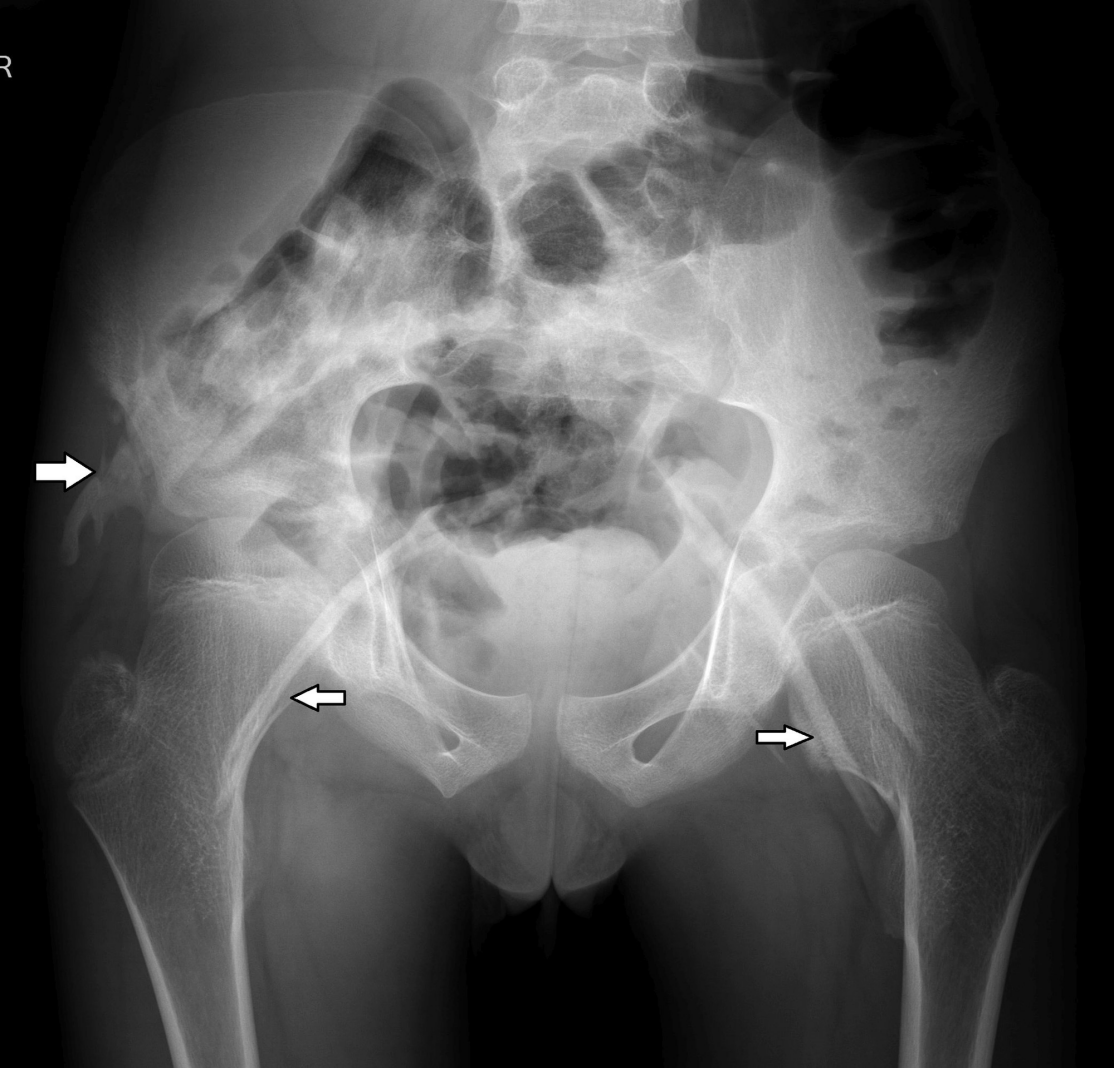
Plain radiograph of pelvis with bilateral hips – anteroposterior view. Broadening of femoral neck with bridge like heterotopic ossifications extending across both the hip joints as well as lateral to the right iliac bone (white arrows).

Radiograph of the pelvis with bilateral hips revealed broadening of femoral neck with bridge-like heterotopic ossifications extending across both the hip joints as well as lateral to the right iliac bone (Figure 7).

**Figure 8:**
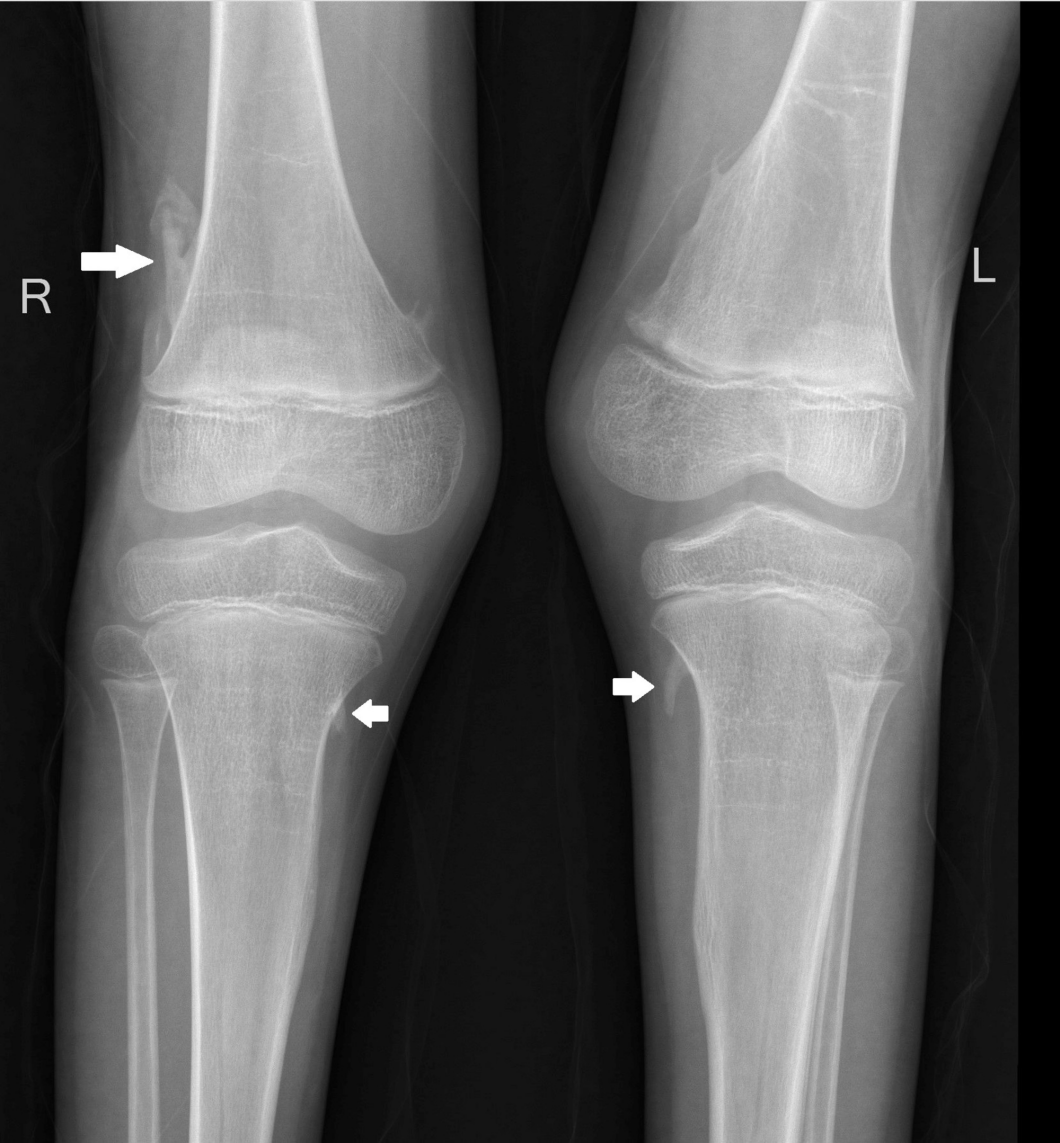
Plain radiograph of bilateral knees – anteroposterior view. Bony outgrowth like appearance due to ossification along ligamentous insertion on right lateral distal femoral metaphysis as well as bilateral proximal medial tibial metaphyses producing pseudoexostoses (white arrows).

Plain radiographs of the knees showed bony outgrowth like appearance due to ossification along ligamentous insertion on right lateral distal femoral metaphyses as well as bilateral proximal medial tibial metaphyses producing pseudoexostoses (Figure 8).

**Figure 9:**
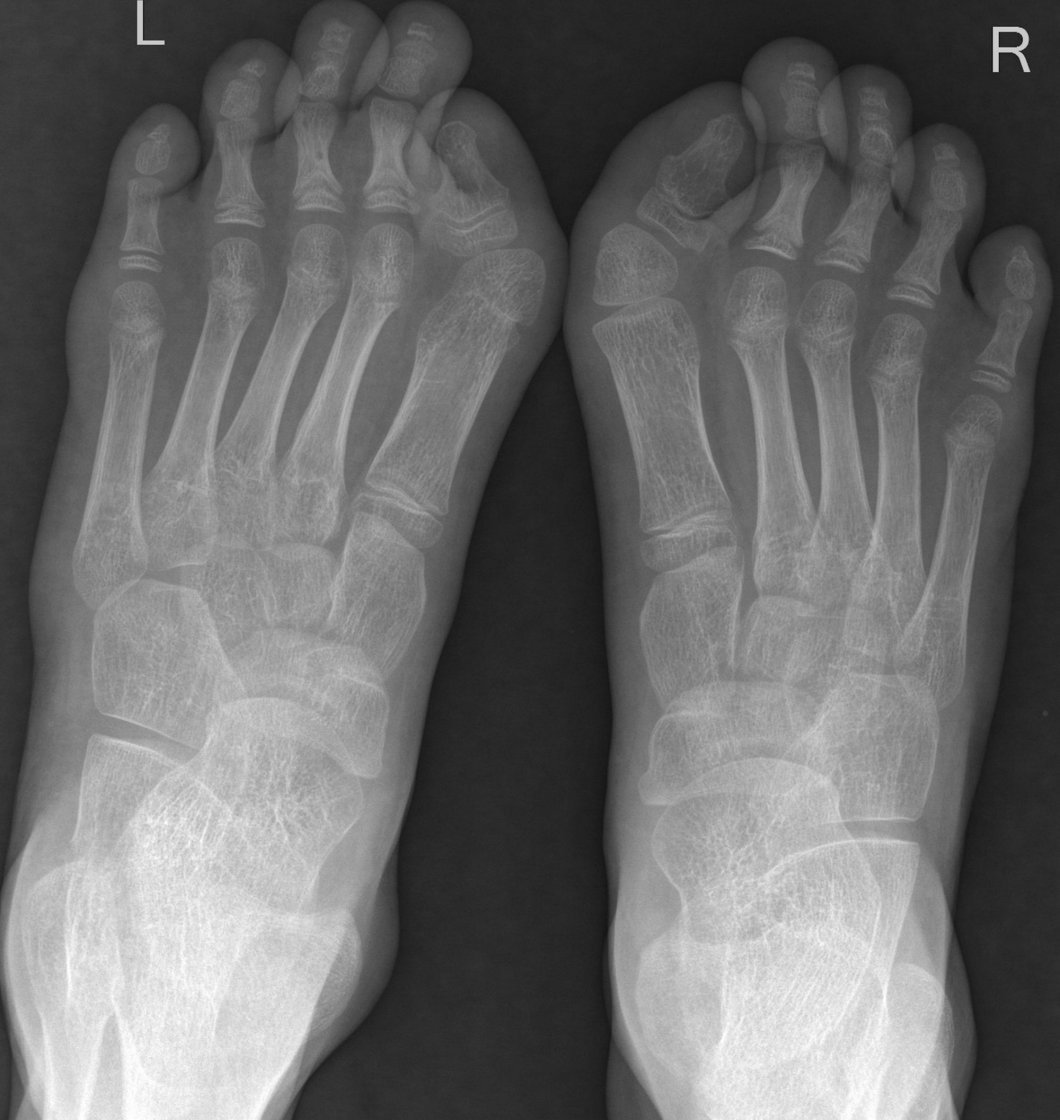
Plain radiograph of both foot – anteroposterior view. Bilateral hallux valgus with monophalangism of the great toes.

Radiographs of both the foot revealed bilateral hallux valgus with monophalangism of the great toes (Figure 9).

Based on the presence of congenital bilateral great toe anomalies and progressive widespread heterotopic ossification in the characteristic anatomic pattern, a clinical diagnosis of FOP was arrived at. The patient’s relatives were counselled about the prognosis and lack of definite treatment and cure for the disease. They were also informed about the presence of support groups like International Fibrodysplasia Ossificans Progressiva Association (IFOPA) to advocate, support and connect patients, and their families, afflicted with this debilitating disorder.

## Discussion

FOP is a debilitating disease of progressive heterotopic bone formation in the extraskeletal tissues [1]. There are approximately 800 cases reported of this rare genetic disorder worldwide [6]. It has been reported from all parts of the world without any ethnic, racial, geographical or gender predisposition; however, a higher prevalence is reported from France [7]. Although it is characterized by spontaneous new mutations in the majority of the cases, familial cases have also been described with an autosomal dominant inheritance [8]. The symptoms typically begin in the first decade of life at a mean age of 7.1 years with a characteristic temporal and spatial pattern of episodic, painful, inflammatory soft tissue swellings [6]. The classical phenotype of FOP has two distinct physical features: formation of extraskeletal ossified masses in a progressive and episodic manner (flare-ups) following a particular anatomic pattern and presence of congenital hallux valgus with microdactyly. These flare-ups are typically first seen in the dorsal, proximal and cranial regions of the body and then involve the caudal, ventral and distal parts. As more and more muscles, ligaments and tendons get ossified, severe cumulative disability ensues by severely restricting the range of motion of various joints [1]. These patients generally become wheelchair-bound by the third decade and death occurs in the fourth decade due to cardio-respiratory complications [9].

The flare-ups of FOP can be triggered by various events like minor trauma, intramuscular injections, certain viral illnesses, as well as any surgical procedures like biopsy [10]. Therefore, before a diagnostic procedure like a biopsy is contemplated in any suspicious extraskeletal ossified mass, features of FOP like congenital malformed great toes should be looked upon. In our case, surgical removal of the first swelling triggered a rapid development of various other soft tissue ossified masses, a series of events which could have been avoided by clinically ruling out FOP first. Apart from malformed great toes, various other anomalies can be observed in these patients like clinodactyly, thumb malformations, inverted nipples, broad short femoral neck, increased prevalence of kidney stones, cervical spine fusions and conductive hearing loss [11, 12]. Our patient did not have any such anomaly besides restriction of neck flexion and extension movements. In FOP, there is sparing of cardiac muscles, smooth muscles as well as certain skeletal muscle groups like diaphragm, tongue and extraocular muscles [1].

The genetic change identified in all the classic cases of FOP includes a missense mutation in the glycine-serine activation domain of the gene Activin receptor-like kinase 2 (ACVR1, ALK2), a bone morphogenetic protein (BMP) type I receptor [13]. This recent genetic breakthrough of increased activation of BMP signalling pathway as the cause of progressive HEO has opened the doors for a series of research trials, in order to find a therapeutic solution for this dreaded disease. The characteristic lesions of FOP have been described to undergo a transition from an initial inflammatory stage (painful) to an intense fibroproliferative stage, which further changes to a chondrogenic and finally an osteogenic stage; a particular active lesion contains areas of all the stages [1]. In our patient, the inflammatory stage was not seen as the lesions were never associated with pain, a feature which could have created a diagnostic dilemma had it not been for the presence of malformed toes. Recently, a cumulative analogue joint involvement scale (CAJIS) has been described for objective assessment of the joint mobility limitation in these patients [14]. On the basis of joint dysfunction and its consequences, five clinical stages have been described for FOP: early/mild, moderate, late/severe, profound and end-stage disease [15].

The diagnosis of FOP is mainly clinical based on the specific pattern of physical findings including progressive HEO in a particular anatomic pattern with the presence of malformed great toes. Plain radiographs and computed tomography (CT) help in better delineation of the FOP lesions, demonstrating sheets, plates and ribbons of heterotopic bone formation [16]. The routine biochemical laboratory investigations are normal in such patients. Genetic analysis can provide confirmation of the diagnosis in case of atypical findings and doubtful phenotypic features [10]. At present, there is no cure for FOP and the current therapeutic strategies focus on early diagnosis and preventive actions in order to delay and avoid the formation of the characteristic lesions. The various treatment options described to control the flare-ups include high dose corticosteroids, bisphosphonates and non-steroidal anti-inflammatory drugs [6]. After the breakthrough discovery of the genetic basis of FOP, research trials have begun targeting various potential aspects and areas of the BMP signalling pathway, and various cell models are being prepared. The current research strategies are targeting dysregulated BMP signalling with agents like Dorsomorphin, Perhexiline and Imatinib mesylate; neofunction of the mutated ACVR1 receptor with anti-ActA antibodies and Rapamycin; the chondrogenetic differentiation processes with retinoic acid receptor gamma agonists like Palovarotene; the expression of the mutated receptor; the immune system with corticosteroids; as well as the local microenvironment of the lesions [17-19]. Although there has been significant progress in basic and translational research in FOP in the recent past, a lot of knowledge is yet to be unearthed. The need of the hour is to encourage high-level research to foster the development of furthermore therapeutic options for FOP patients so that they can prevent the devastating consequences of this disabling disease.

## Conclusions

FOP is a rare and crippling genetic disease characterized by areas of abnormal bone formation in muscles, ligaments, tendons and joint capsules. The typical presentation involves repeated episodes of painful inflammatory soft tissue swellings beginning in the first decade of life, which later lead to the formation of hard bony masses. Our case did not have any prior complaints of painful soft tissue lesions or the characteristic flare-ups of the disease ever, and presented with painless hard bony masses with associated disability due to the formation of joint contractures. In such atypical cases, the specific pattern of lesions with its associated disability due to the formation of joint contractures, along with the typical great toe malformations can help in clinching the correct diagnosis of FOP. Also, inadvertent surgical removal of the first soft tissue swelling resulted in a rapid evolution of new lesions in our patient. Surgical procedures including biopsies should be strictly avoided in such patients to prevent triggering the development of newer lesions.

## References

[1] Pignolo RJ, Shore EM, Kaplan FS (2013). Fibrodysplasia ossificans progressiva: diagnosis, management, and therapeutic horizons. Pediatr Endocrinol Rev.

[2] Kartal-Kaess M, Shore EM, Xu M (2010). Fibrodysplasia ossificans progressiva (FOP): watch the great toes!. Eur J Pediatr.

[3] Ram GG, K KA, Vijayaraghavan PV (2015). The stone women-Myositis ossificans Progressiva. J Orthop Case Rep.

[4] Hasegawa S, Victoria T, Kayserili H (2016). Characteristic calcaneal ossification: an additional early radiographic finding in infants with fibrodysplasia ossificans progressiva. Pediatr Radiol.

[5] Qi Z, Luan J, Zhou X, Cui Y, Han J (2017). Fibrodysplasia ossificans progressiva: basic understanding and experimental models. Intractable Rare Dis Res.

[6] Pachajoa H, Botero AF (2015). Clinical and molecular characterisation of two siblings with fibrodysplasia ossificans progressiva, from the Colombian Pacific coast (South America). BMJ Case Rep.

[7] Baujat G, Choquet R, Bouée S (2017). Prevalence of fibrodysplasia ossificans progressiva (FOP) in France: an estimate based on a record linkage of two national databases. Orphanet J Rare Dis.

[8] Kaplan FS, Pignolo RJ, Al Mukaddam MM, Shore EM (2017). Hard targets for a second skeleton: therapeutic horizons for fibrodysplasia ossificans progressiva (FOP). Expert Opin Orphan Drugs.

[9] Kaplan FS, Zasloff MA, Kitterman JA, Shore EM, Hong CC, Rocke DM (2010). Early mortality and cardiorespiratory failure in patients with fibrodysplasia ossificans progressiva. J Bone Joint Surg Am.

[10] Tian S, Zhu J, Lu Y (2018). Difficult diagnosis and genetic analysis of fibrodysplasia ossificans progressiva: a case report. BMC Med Genet.

[11] Singh A, Pradhan G, Kumari C, Kapoor S (2016). Early recognition of fibrodysplasia ossificans progressiva-important for the clinician. J Nepal Med Assoc.

[12] Gupta RR, Delai PLR, Glaser DL, Rocke DM, Al Mukaddam M, Pignolo RJ, Kaplan FS (2018). Prevalence and risk factors for kidney stones in fibrodysplasia ossificans progressiva. Bone.

[13] Sanchez-Duffhues G, de Gorter DJ, Ten Dijke P (2016). Towards a cure for Fibrodysplasia ossificans progressiva. Ann Transl Med.

[14] Kaplan FS, Al Mukaddam M, Pignolo RJ (2017). A cumulative analogue joint involvement scale (CAJIS) for fibrodysplasia ossificans progressiva (FOP). Bone.

[15] Pignolo RJ, Kaplan FS (2018). Clinical staging of Fibrodysplasia Ossificans Progressiva (FOP). Bone.

[16] Mukaddam MA, Rajapakse CS, Pignolo RJ, Kaplan FS, Smith SE (2018). Imaging assessment of fibrodysplasia ossificans progressiva: qualitative, quantitative and questionable. Bone.

[17] Cappato S, Giacopelli F, Ravazzolo R, Bocciardi R (2018). The horizon of a therapy for rare genetic diseases: a “druggable” future for fibrodysplasia ossificans progressiva. Int J Mol Sci.

[18] Kaplan FS, Andolina JR, Adamson PC (2018). Early clinical observations on the use of imatinib mesylate in FOP: a report of seven cases. Bone.

[19] Kaplan FS, Zeitlin L, Dunn SP, Benor S, Hagin D, Al Mukaddam M, Pignolo RJ (2018). Acute and chronic rapamycin use in patients with fibrodysplasia ossificans progressiva: a report of two cases. Bone.

